# Intereye comparison of ocular factors in normal tension glaucoma with asymmetric visual field loss in Korean population

**DOI:** 10.1371/journal.pone.0186236

**Published:** 2017-10-17

**Authors:** Eun Jung Lee, Jong Chul Han, Changwon Kee

**Affiliations:** Department of Ophthalmology, Samsung Medical Center, Sungkyunkwan University School of Medicine, Seoul, Korea; Bascom Palmer Eye Institute, UNITED STATES

## Abstract

**Purpose:**

To identify ocular parameters corresponding to asymmetric visual field (VF) loss in normal tension glaucoma (NTG) through intereye comparisons.

**Patients and methods:**

Medical records of NTG patients with asymmetric and symmetric VF losses were retrospectively reviewed. The criterion for asymmetry in VF was 6 dB difference of mean deviation. Refractive error, intraocular pressure (IOP), central corneal thickness, ovality index, and peripapillary atrophy (PPA)/disc area ratio were obtained from each patient. Intereye comparison was performed for asymmetric group, symmetric group, and myopic and nonmyopic asymmetric subgroups.

**Results:**

We included 155 patients; 110 patients in asymmetric group and 45 patients in symmetric group. In intereye comparison for total asymmetric group, refractive error (*P* = 0.006), initial IOP (*P* = 0.001), ovality index (*P* = 0.008), and PPA (*P* < 0.001) were significantly asymmetric. For myopic subgroup, refractive error (*P* = 0.004), ovality index (*P* = 0.001), and PPA (*P* = 0.003) were significant factors. For nonmyopic subgroup, initial IOP (*P* = 0.003) and PPA (*P* = 0.007) were significant factors. Symmetric group showed no significant difference between the eyes. Multivariate analysis demonstrated that refractive error (*P* = 0.002) and PPA (*P* = 0.028) were significant factors in myopic subgroup, and initial IOP (*P* = 0.022) and PPA (*P* = 0.002) were significant factors in nonmyopic subgroup.

**Conclusions:**

In this intereye comparison, the more myopic eye in myopic NTG patient, and the more pressured eye in nonmyopic NTG patient demonstrated more severe VF loss. Myopic and nonmyopic patients may follow different pathophysiologic processes. Discriminative attentions should be paid to NTG patients by subtypes.

## Introduction

Normal tension glaucoma (NTG) is defined as chronic open-angle glaucoma with a progressive visual field (VF) defect and optic nerve damage, where untreated intraocular pressure (IOP) is within statistically normal range.[1] Although VF loss generally progresses slowly, some cases progress more rapidly,[2] and IOP-lowering treatment does not always prevent the progression.[3] Identification of rapid progressors would be practically beneficial.

If an ocular parameter with significant difference can be identified between the eyes in an asymmetric pair of already established diagnosis of glaucoma, it would reflect a relatively faster deterioration rate of glaucomatous damage. It may further help predict the risk of future progression by the parameter, while the pattern of such relationship is not known; it can be linear, or be associated with certain threshold values for significantly more aggressive damages. Moreover, intereye comparison of a single subject enables an analysis free of systemic confounding factors including systemic diseases, race, gender, and age, which is critical when evaluating progressive disease such as glaucoma.

Parameters for progression of open-angle glaucoma from clinical trials include older age, higher IOP at baseline, larger IOP fluctuations, thinner central corneal thickness (CCT), worse initial VF status, presence of migraines, and presence of disc hemorrhage.[4–8] Collaborative normal tension glaucoma study demonstrated that IOP lowering, female gender, history of migraine, and the presence of disc hemorrhage affected glaucoma progression in NTG patients.[8] Treated IOP in NTG,[9–11] degree of myopia in primary open-angle glaucoma (POAG) and NTG,[12–16] corneal biomechanical properties such as corneal hysteresis,[17] β-zone peripapillary atrophy (PPA) in POAG and NTG,[18–23] and β-zone microstructure in NTG,[24] are also associated with glaucoma progression.

Risk factors for progression can differ by the type of glaucoma, for example, POAG and NTG. In NTG, we could observe both positive and negative results on the relationship between glaucoma progression and IOP, CCT, and myopic degrees.[10,25–35] The role of increased IOP in glaucomatous damage seems concrete, even in NTG, although it may not be the only factor. These inconsistent results would thus imply that non-IOP factors would affect glaucoma progression, especially in NTG patients.

Especially, presence and degree of myopia might be noted separately in evaluation of such risk factors. Myopic optic disc changes can affect the pathogenesis of glaucoma, although the exact mechanism remains elusive.[36] In the literature, the results from many studies are also inconclusive. Myopia was mentioned as a risk factor in the progression in POAG.[12,14,37] Axial myopia was associated with progression in open-angle glaucoma.[15] In studies with NTG patients in Japanese population, myopia was a positive factor for glaucoma progression.[27,34,35] Meanwhile, there are other studies reporting absence of difference in glaucoma progression between myopic and nonmyopic group.[16,27,32,38]

In our study, we recruited a group of NTG patients with marked VF asymmetry to perform the intereye comparison for ocular factors associated with asymmetric severity. We also recruited a group of patients with no VF differences for contrast, and investigated if clinical parameters differ between asymmetric versus symmetric NTG patients.

## Materials and methods

### Study subjects

This was a retrospective, cross-sectional study. The medical records of bilateral NTG patients who visited Samsung Medical Center (Seoul, Korea) between March, 2010 and August, 2016 were reviewed. This study followed all guidelines for experimental investigation in human subjects, was approved by the Samsung Medical Center Institutional Review Board, and adhered to the tenets of the Declaration of Helsinki.

Each patient underwent a comprehensive ophthalmic examination including measurement of visual acuity and refractive error, Goldmann applanation tonometry, slit-lamp biomicroscopy, gonioscopic examination, dilated stereoscopic examination of the optic nerve head, color and red-free fundus photography (Topcon, Paramus, NJ, USA), static automated perimetry using the central 30–2 Humphrey Field Analyzer (HFA model 640 or model 740; Humphrey Instruments Inc., San Leandro, CA, USA), ultrasound pachymetry (Tomey SP-3000, Tomey Ltd., Nagoya, Japan), and spectral-domain optical coherence tomography (OCT) with Cirrus HD-OCT (Carl Zeiss Meditec, Dublin, CA).

Diagnosis of NTG was based on: (1) untreated baseline IOP ≤ 21 mmHg, (2) typical glaucomatous optic disc change, (3) reproducible glaucomatous VF defect, and (4) open angles on gonioscopy. A glaucomatous VF was defined as glaucoma hemifield test results outside normal limits on at least 2 consecutive baseline VF tests and the presence of at least 3 contiguous test points on the pattern standard deviation plot at *P* < 5%, with at least 1 at *P* < 1%, excluding points on the edge of the field or those directly above and below the blind spot. A reliable VF test had to fulfill 3 criteria: fixation loss less than 20% and false-positive and false-negative rates of less than 15%. The location and pattern of the defect had to be consistent between the 2 consecutive VF examinations, and the glaucomatous optic disc damage had to be consistent with the VF abnormality. Untreated baseline IOP was defined as the average IOP of two consecutive visits in the absence of IOP-lowering medication. In patients using IOP medications, IOP was measured after discontinuing the medications for 4 weeks.

Patients were classified into an asymmetric or symmetric NTG group by the mean deviation (MD) values from VF tests. The better eye and worse eye were defined according to their MD values. Asymmetric NTG was defined as a MD difference of at least 6 dB. Our criteria of 6dB was based on AGIS staging system,[39] which is a unit dividing the stages. The asymmetricity had to be consistent, not transient VF deteriorations as fluctuation.

Symmetric NTG was defined as a MD difference less than 3 dB, a cutoff value to consider the fluctuation effect.[40] Because eyes with early-stage damage have possibility of various future disease course, final relative degree of VF defects might progress to be opposite from the point of enrollment. Therefore, patient with better eye MD not worse than -6dB was excluded.

Two independent ophthalmologists (JCH and EJL) decided on the asymmetricity. In cases of reader disagreement, consensus was reached through discussion. We excluded cases that could not reach an agreement.

We also excluded following cases; (1) eyes with media opacities including cataract of LOCS III grade more than C2, N2 or P2; (2) asymmetric grade of cataract between the eyes, (3) history of unilateral cataract surgery; (4) systemic disease that can affect VF tests; (5) severe myopic degeneration or retinal disease; (6) nonglaucomatous optic neuropathy or other neuro-ophthalmic disease; (7) possibility of secondary glaucoma including inflammatory eye disease, pseudoexfoliation, or pigment dispersion, being younger than 30 years old at the time of diagnosis; (8) diabetic retinopathy or other retinal disease as venous obstruction; (9) history of trauma or intraocular surgery other than uneventful bilateral cataract surgery performed in our clinic with stable IOP; and (10) previous corneal refractive surgery.

For subgroup analysis, patients were divided into a myopic and a nonmyopic group. The myopic group had refractive error of -1.0 diopters (D) or less in the worse eye, and the nonmyopic group had refractive error between -1.0 D and +1.0 D in the worse eye.

Unilateral NTG patients were excluded. Also, bilateral NTG patients who had diagnosis of NTG in one eye first, and then in the fellow eye with an interval period of longer than 2 years were excluded.

### Measurement of optic nerve head (ONH) parameters

The ovality index was defined as the ratio between the longest and shortest diameters of the optic disc.[41] Optic disc torsion was defined as the deviation of the long axis of the optic disc from the vertical line drawn at 90° from the fovea—clinical optic disc center axis. Absolute values of measured torsion were used, and the unit of measurement was degrees. PPA was defined as inner crescent of chorioretinal atrophy with visible sclera and choroidal vessels, excluding alpha zone.[18] PPA/disc area ratios were calculated by plotting the outline PPA and clinical disc by using a cursor to trace the disc and PPA margins onto the image.[42] The pixel areas of the PPA and clinical disc were used to calculate the ratio of each areas. Due to a large proportion of patients with myopic tilted discs, we did not perform cup-to-disc ratio description for comparison of ocular parameters. Images were evaluated using color fundus photographs with Image J software (version 1.52; National Institutes of Health, Bethesda, MD, USA).

### Statistical analysis

The χ^2^ test was used to compare categorical data, and independent t-test was used for comparison of parameters between the two groups. Paired t-test was used for intereye comparison of parameters. To investigate correlations between parameters, we performed Spearman correlation analysis. Multivariate analysis was performed by the generalized estimation equation approach, with worse eye as a binary outcome measure. All statistical analyses were performed with SPSS software version 22.0 (SPSS, Inc., Chicago, IL, USA). A *P* value less than 0.05 was considered statistically significant.

## Results

Among the 239 patients meeting the VF criteria, 47 patients were excluded. We excluded patients with corneal opacity (2), retinal diseases including venous obstruction and diabetic retinopathy (12), superior segmental optic nerve hypoplasia (4), and possible secondary glaucoma (7). Patients with previous outside cataract surgery (3), unilateral cataract surgery (1), and refractive surgery (3) were also excluded. Lastly, unilateral or sequential NTG diagnosis (18), unreliable VF and asymmetricity nonconsensus (14) as well as being younger than 30 years old (4) were excluded. Additionally, patients with hyperopia above +1.0 D (11) were excluded.

At the first diagnosis of NTG, the patients received betaxolol 5 mg/mL (Alcon, Inc, Fort Worth, Texas, USA) twice daily. During the follow-up, if glaucoma progressed or no satisfactory IOP lowering was observed, betaxolol was replaced by latanoprost 50 μg/mL (Pfizer, Inc., New York, New York, USA). According to the patient’s status, addition or replacement with brimonidine 2 mg/mL (Allergan, Inc., Irvine, California, USA) or fixed-combination of brinzolamide 1%/brimonidine 0.2% was performed.

Finally, 155 patients were analyzed; 110 patients were in the asymmetric group, and 45 patients were in the symmetric group. Mean age of asymmetric group was younger than symmetric group (*P* = 0.016). No other parameters differed between the two groups. In the asymmetric group, mean age of myopic subgroup was significantly younger than nonmyopic subgroup (48.7 ± 10.1 vs. 61.6 ± 10.4, *P* < 0.001), and hypertension was more prevalent in the nonmyopic subgroup (*P* = 0.005, Table 1). In intereye comparison for asymmetric group, refractive error (P = 0.006), initial IOP (*P* = 0.001), ovality index (*P* = 0.008), and PPA (*P* < 0.001) were significantly different between the eyes. Different results were observed according to the subgroups of the asymmetric group. For myopic subgroup, refractive error (*P* = 0.004), ovality index (*P* = 0.001), and PPA (*P* = 0.003) were significant factors. IOP showed a trend for corresponding asymmetry, but did not reach statistical significance (*P* = 0.072). For nonmyopic subgroup, initial IOP (*P* = 0.003) and PPA (*P* = 0.007) were only significant factors, while refractive error showed only marginal significance (*P* = 0.080). No parameter exhibited significant difference in the symmetric NTG group (Table 2).

**Table 1:** Patient characteristics in the asymmetric and symmetric groups.

	Asymmetric group	Myopic subgroup	Nonmyopic subgroup	Symmetric group	P*	*P*^†^
Number of patients, n	110	66	44	45		
Age at diagnosis (years)	53.9 ± 12.0 (30–80)	48.7 ± 10.1 (30–76)	61.6 ± 10.4 (30–80)	59.3 ± 13.5 (35–88)	0.016	<0.001
Sex, male/female	70/40	45/21	25/19	26/19	0.585	0.234
Hypertension, yes/no	20/90	6/60	14/30	12/33	0.276	0.005
Diabetes, yes/no	12/98	7/59	5/39	5/40	1	1
Worse eye laterality, right/left	56/54	29/37	25/19	25/20	0.723	0.243
Follow-up period (months)	59.05 ± 48.53	54.89 ± 49.26	65.20 ± 46.71	64.71 ± 49.45	0.517	0.286

dB, decibel; D, diopters; IOP, intraocular pressure; PPA, peripapillary atrophy;

Unless otherwise indicated, data are given as the mean ± SD (range).

* *P* value was calculated between total asymmetric group and symmetric group, by independent t-test for continuous variables, and χ^2^ test for categorical variables.

^†^ *P* value was calculated between myopic and nonmyopic asymmetric group, by independent t-test for continuous variables, and χ^2^ test for categorical variables.

**Table 2:** Intereye comparison of ocular parameters in the asymmetric and symmetric groups.

	Worse eye	Better eye	P*
**Asymmetric group**			
Number of eyes, n	110	110	NA
Mean deviation, dB	-14.07 ± 4.76 (-31.10 – -6.21)	-4.01 ± 3.02 (-12.17 – 2.41)	<0.001
Spherical equivalent, D	-3.05 ± 3.27 (-9.50 – +1.0)	-2.78 ± 3.17 (-10.00 – +1.0)	0.006
Untreated initial IOP, mmHg	16.52 ± 2.37	16.03 ± 2.41	0.001
Central corneal thickness, μm	536.3 ± 32.7	537.3 ± 32.7	0.292
Ovality index	1.211 ± 0.172	1.178 ± 0.146	0.008
Torsion, degree	14.11 ± 16.25	14.49 ± 17.12	0.768
PPA/disc area ratio	0.664 ± 0.410	0.538 ± 0.395	<0.001
**Myopic asymmetric group**			
Number of eyes, n	66	66	NA
Mean deviation, dB	-14.14 ± 4.63 (-28.29 – -6.21)	-4.26 ± 3.05 (-11.51 – 0.88)	<0.001
Spherical equivalent, D	-5.13 ± 2.43 (-9.50 – -1.38)	-4.77 ± 2.52 (-10.00 – +0.38)	0.004
Untreated initial IOP, mmHg	16.70 ± 2.31	16.42 ± 2.19	0.072
Central corneal thickness, μm	540.3 ± 29.0	541.4 ± 28.0	0.35
Ovality index	1.274 ± 0.185	1.215 ± 0.170	0.001
Torsion, degree	14.37 ± 17.05	16.35 ± 18.60	0.241
PPA/disc area ratio	0.773 ± 0.441	0.641 ± 0.458	0.003
**Nonmyopic asymmetric group**			
Number of eyes, n	44	44	NA
Mean deviation, dB	-14.00 ± 5.07 (-31.10 – -7.52)	-3.61 ± 2.94 (-10.69 – 2.41)	<0.001
Spherical equivalent, D	+0.26 ± 0.54 (-0.75 – +1.00)	+0.14 ± 0.74 (-2.00 – +1.00)	0.080
Untreated initial IOP, mmHg	16.23 ± 2.47	15.41 ± 2.64	0.003
Central corneal thickness, μm	530.2 ± 37.1	531.2 ± 38.2	0.584
Ovality index	1.115 ± 0.083	1.125 ± 0.070	0.318
Torsion, degree	13.73 ± 15.17	11.70 ± 14.40	0.298
PPA/disc area ratio	0.499 ± 0.291	0.384 ± 0.196	0.007
**Symmetric group**			
Number of eyes, n	45	45	NA
Mean deviation, dB	-15.08 ± 6.95 (-29.89 – -6.77)	-14.02 ± 6.81 (-29.33 – -6.07)	<0.001
Spherical equivalent, D	-2.96 ± 2.91 (-10.50 – +1.00)	-2.88 ± 2.76 (-10.00 – +1.00)	0.561
Untreated initial IOP, mmHg	16.03 ± 2.27	15.95 ± 2.40	0.719
Central corneal thickness, μm	531.0 ± 29.6	531.7 ± 30.4	0.619
Ovality index	1.182 ± 0.111	1.190± 0.129	0.600
Torsion, degree	15.80 ± 24.38	12.69 ± 17.49	0.252
PPA/disc area ratio	0.719 ± 0.434	0.770 ± 0.676	0.449

NA, not applicable; dB, decibel; D, diopters; IOP, intraocular pressure; PPA, peripapillary atrophy;

Unless otherwise indicated, data are given as the mean ± SD (range).

* *P* value was calculated by paired t-test.

In correlation analysis for myopic subgroup, refractive error was significantly associated with ovality index and PPA (both *P* < 0.001), and those were not included in the multivariate analysis model at the same time because of close relationship between those parameters could confound the statistical results. In nonmyopic subgroup, refractive error and PPA were not correlated (*P* = 0.495), and refractive error and IOP were slightly correlated (*P* = 0.011, Table 3). Therefore, for the same reason as in myopic subgroup, models with and without refractive error were conducted for multivariate analysis.

**Table 3:** Correlation analysis between parameters in myopic and nonmyopic asymmetric group.

	IOP	Central corneal thickness	Ovality index	PPA/disc area ratio
Myopic subgroup				
Spherical equivalent	0.047 (0.601)	-0.041 (0.645)	-0.309 (<0.001)	-0.282 (0.001)
IOP		0.126 (0.164)	0.021 (0.816)	0.024 (0.790)
Ovality index	0.021 (0.816)	0.054 (0.546)		0.497 (<0.001)
Nonmyopic subgroup				
Spherical equivalent	0.293 (0.011)	0.020 (0.862)	-0.079 (0.479)	0.076 (0.495)
IOP		0.139 (0.224)	-0.124 (0.274)	0.100 (0.376)
Ovality index	-0.124 (0.274)	0.205 (0.059)		0.213 (0.046)

IOP, intraocular pressure; PPA, peripapillary atrophy;

Data are given as the Spearman’s rho (*P* value).

In multivariate analysis, refractive error (OR, 1.068; 95% CI, 1.024–1.114; *P* = 0.002) and PPA (OR, 0.512; 95% CI, 0.282–0.931; *P* = 0.028) were significant in myopic subgroup. For nonmyopic subgroup, initial IOP (OR, 0.906; 95% CI, 0.830–0.988, *P* = 0.026) and PPA (OR, 0.139; 95% CI, 0.045–0.432, *P* = 0.001) were significant (Table 4).

**Table 4:** Multivariate analysis of intereye comparisons in the myopic and nonmyopic asymmetric group.

	Univariate analysis			Multivariate analysis			Multivariate analysis		
				Model 1			Model 2		
Parameter	Odds ratio	95% CI	P*	Odds ratio	95% CI	P*	Odds ratio	95% CI	P*
**Myopic subgroup**									
Spherical equivalent	1.062	1.021–1.105	0.003	1.068	1.024–1.114	0.002			
Untreated initial IOP	0.945	0.889–1.005	0.073	0.940	0.879–1.005	0.071	0.948	0.886–1.014	0.121
CCT	1.001	0.998–1.004	0.343						
Ovality index	0.149	0.038–0.582	0.006						
Torsion	1.006	0.995–1.018	0.259						
PPA/Disc ratio	0.512	0.300–0.875	0.014				0.512	0.282–0.931	0.028
**Nonmyopic subgroup**									
Spherical equivalent	0.765	0.576–1.016	0.063	0.887	0.601–1.308	0.544			
Untreated initial IOP	0.856	0.776–0.945	0.002	0.869	0.770–0.979	0.022	0.864	0.769–0.970	0.013
CCT	1.001	0.998–1.005	0.396						
Ovality index	0.070	0.001–5.370	0.230						
Torsion	0.999	0.984–1.013	0.848						
PPA/Disc ratio	0.107	0.030–0.391	0.001	0.112	0.028–0.457	0.002	0.110	0.028–0.432	0.002

OR, odds ratio; CI, confidence interval; IOP, intraocular pressure; CCT, central corneal thickness; PPA, peripapillary atrophy;

* *P* value was calculated by generalized estimation equation (GEE) approach, using binary logistic regression with worse versus better eye as binary outcome measurement.

In multivariate analysis for myopic subgroup, we could observe that significance of refractive error was maintained when evaluated simultaneously with untreated initial IOP (OR, 1.068; 95% CI, 1.024–1.114; P = 0.002). Similarly, PPA was also significant (OR, 0.512; 95% CI, 0.282–0.931; P = 0.028). In nonmyopic subgroup, initial IOP (OR, 0.869; 95% CI, 0.770–0.979, *P* = 0.022) and PPA (OR, 0.112; 95% CI, 0.028–0.457, *P* = 0.002) were independently significant, while refractive error was not.

**Fig 1:**
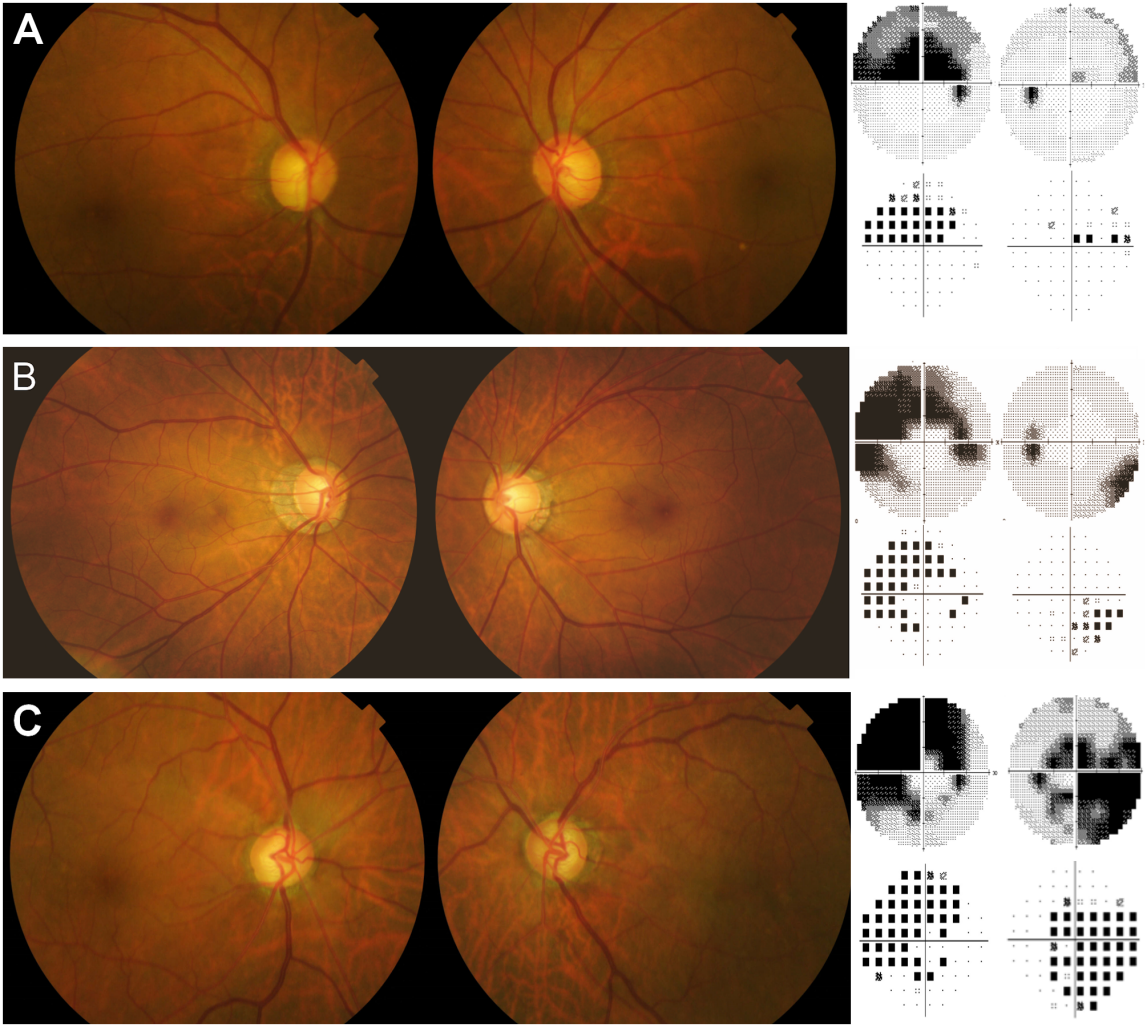
Representative cases from the asymmetric myopic, nonmyopic, and symmetric normal tension glaucoma groups. (A) A 74-year-old male patient with refractive error of +1.25 D = -1.75 D x 90° and +1.25 D = -1.25 D x 90° on the right and the left eye, respectively. Visual field test results showed MD of -8.68 and -0.99 dB, for the right eye and the left eye, respectively. The baseline untreated IOPs were 17 and 14 mmHg, respectively. The ovality indices were similar between the worse eye and the better eye (1.057 and 1.087, respectively). The worse eye demonstrated a slightly larger PPA/disc area ratio than the better eye (0.487 and 0.397, respectively). (B) A 42-year-old male patient with asymmetric myopia of -7.25 diopters (D) = -0.75 D x 180° and -5.75 D = -0.25 D x 90° on the right and the left eye, respectively. The baseline untreated intraocular pressures (IOPs) were 16 and 15 mmHg, respectively. Visual field test results showed mean deviation (MD) of -13.68 dB and -2.90 dB, for the right eye and the left eye, respectively. The ovality index was larger in the worse eye than in the better eye (1.365 and 1.120, respectively). Also, the worse eye showed a larger peripapillary area (PPA)/disc area ratio than the better eye (1.591 and 0.969, respectively). (C) A 59-year-old male patient with visual field defects of similar severity (MD -17.24 dB and -16.49 dB, respectively). The refractive errors were -0.25 D = -2.0 D x 180° and -0.25 D = -1.5 D x 180° for the right and the left eye, respectively. The baseline untreated IOPs were 17 and 16 mmHg, respectively. The ovality indices (1.038 and 1.027) and PPA/disc area ratios (0.304 and 0.396) were similar between the two eyes.

Representative cases from each subgroup are shown in Fig 1.

## Discussion

As mentioned by Coleman and Miglior,[43] risk factors are associated with disease development while prognostic factors are associated with disease progression. However, it also seems reasonable that pathogenic mechanism or role of a parameter on development and progression of any disease would not be divided completely. Meanwhile, on clinical standpoint, and for clarity of description, we would rather specify the parameters in our study as to be interpreted as prognostic factors rather than developmental risk factors. Our patients had bilateral NTG, and ocular parameters differed significantly with corresponding asymmetry in severity. The view designates the parameters investigated in our study as prognostic factors for more rapid deterioration of glaucomatous retinal nerve fiber layer (RNFL) damages.

Most of all, the relationship between increased IOP and glaucomatous optic nerve damage seems undoubtful, more certainly in case of elevated pressure. And in relative high pressures within statistically normal range, the role of IOP would highly likely exist. The effect of IOP has been demonstrated through many studies. [3–5,9–11,14,22,44,45] Nevertheless, IOP cannot be a single actor for glaucoma, and when the magnitude of IOP elevation is relatively not high, possible role of non-IOP factors in the pathogenesis of glaucoma increases. Intereye studies regarding NTG on the role of IOP is not consistent, and may corroborate this possibility. In NTG patients, early studies revealed higher IOP in worse VF eyes,[21,46,47] while a clinical trial, Low-Pressure Glaucoma Treatment Study (LPGTS) reached a different conclusion.[48] In other words, it denotes that some subpopulation of patients would also be significantly influenced by factors other than IOP. Besides, several other points should also be considered. The method of statistical analysis varied substantially including contingency tables and direct comparisons, and the definition of asymmetry in VF and IOP were mild.[21,46–48] In those studies, criteria for IOP difference was 1 mmHg, and MD difference was 1 to 2 dB.[21,46,47] We could find a few studies employing large MD differences with positive results on IOP-VF asymmetry.[49,50] We speculated that the large VF asymmetry led us to observe significantly higher IOP in the worse eye in nonmyopic group. Moreover, patients with IOP asymmetry over 4 mmHg were excluded in LPGTS.[48] Ruling out eyes with large IOP difference could obscure the results.

In the same point of view, special notes should be placed on myopic group, because they demonstrate distinct structural deformation around the ONH, which might be related to glaucoma pathogenesis. Non-IOP related factors including alterations in the ONH structures such as LC configuration might play larger role in NTG than in POAG in the glaucoma pathogenesis and progression. The specific mechanism has not been proved yet, but suggested candidates include altered biomechanical properties of the lamina cribrosa (LC) and peripapillary sclera.[51] LC and peripapillary scleral thinning,[52] scleral canal tilt,[41] and LC defects associated with glaucoma progression including NTG and other types of glaucoma [53–56] have been reported.

Correspondingly, t myopic NTG and nonmyopic NTG showed different intereye characteristics in our study. This suggest that myopic NTG might have different pathophysiology from nonmyopic counterpart; being myopic might alter components of susceptibility in RNFL damage. Our study confirms that some anatomic or physiologic element associated with myopia, distorted disc anatomy (including PPA) and altered physiology, affect susceptibility to glaucoma, but details of the mechanism remain to be discovered.

This is not a longitudinal study, and we should not expand the interpretation of the results from this analysis to cause-and-effect relationship. Literature shows nonsignificant effect of myopia on NTG,[16,26,27,29,32,34] and even protective role in some studies.[15,16,34,35] Caution is needed in a perspective that the progressive course of myopic NTG might differ from nonmyopic NTG. It has been suggested that RNFL defects in myopia might not progress further.[38,54,57] A subgroup of myopic NTG patients might have a ceiling in RNFL progression to an extent determined by the myopic degree, in contrast to typical NTG where RNFL damage continuously deteriorates to total blindness. Also, selection of myopic glaucoma patients at different stages of progressive course can affect the results. From this cross-sectional design, we can only suspect that more myopia would make the eye to be the more susceptible to glaucomatous damage.

What is not determined is the relative levels of contribution to glaucomatous process of IOP and non-IOP related factors. This unresolved aspect might be influenced by nonsignificant but considerable trends for role of IOP in myopic eyes and refractive error in nonmyopic eyes, respectively. Meanwhile, there is no such ONH deformation in nonmyopic eyes. Thus, we speculated refractive error between emmetropia and mild hyperopia would not significantly influence glaucoma pathogenesis and progression in nonmyopic group.

In our study, we could observe that in more severely damaged eye, PPA was larger. However, this does not suggest that PPA would be the cause of more severe glaucomatous damage. Also, there is not enough evidence to hypothesize that PPA have enlarged along the progressive glaucomatous RNFL damage. Studies are lacking for explanation of related specific mechanism for PPA and glaucoma, and it is not rare to observe progressive glaucoma cases without presence or changes in PPA. Nevertheless, the relationship between PPA and glaucoma has been continuously investigated and demonstrated, in histologic studies including NTG,[58–61] cross-sectional studies, and longitudinal studies in both POAG and NTG.[18–20,22,23,26,58,60–64] Studies demonstrated that PPA was a negative prognostic factor for glaucoma progression, especially in NTG.[10,18,22] Jonas et al suggested that a large area of β-zone PPA is a predictive factor for worsening of glaucoma in ocular hypertension and chronic open-angle glaucoma.[65,66] Of these investigations, we might find important implications from recently introduced classification of PPA microstructures using OCT measurements. PPA were divided into areas with and without Bruch’s membrane (BM).[67,68] For example, γ-zone PPA without BM was associated with myopia while β-zone PPA with intact BM and no RPE correlated with glaucoma. In NTG population, VF progression differed per the presence of the β-zone PPA.[24] Detailed division of PPA microstructures will further facilitate our understanding of PPA with future investigations. What we could observe was that the worse eyes had larger PPA, and the effect was independent of IOP. In addition, advanced imaging with OCT is not currently widely available. Thus, we suggest that it would be advisable to pay attention to distinctly asymmetric PPA between the two eyes and place possibility of more rapid future progression in corresponding manner.

Ovality index was associated with refractive error and we speculated that it was the result of myopic changes. In myopic eyes, tilting of the ONH occurs with eyeball elongation and we could expect with more eyeball elongation, more tilt would be observed, thus leading to higher ovality index. Nevertheless, it cannot represent the exact geometry of ONH structures as it was calculated from simple ratio of superficial clinical disc margins from plain photographs. A more complex configuration exists including the deep ONH such as LC and relationship with surrounding anatomical components, and these should be delicately considered. Therefore, we regarded ovality index as an additive parameter that would at least provide robust estimation for degree of ONH deformation, and placed no further importance in our analysis.

Role of CCT was weak in our study. Investigators have focused on the role of CCT in glaucoma because of the possible relationships between the sclera, lamina, and cornea. Thinner CCT was associated with the state of glaucoma in several studies,[17,69–73] whereas conflicting evidences exist including POAG and NTG.[74–77] Recently, CCT was associated with the state of glaucoma damage while corneal hysteresis was related to the progression.[17] We speculate no specific reason that CCT would directly influence the NTG progression, but further studies including corneal hysteresis measurements are warranted.

For the age difference, we considered that increasing trend for ocular examination in myopic subjects for refractive surgery would have led to the younger age in the myopic group. Or the myopic RNFL damage could differ in pathophysiology from pure NTG, and thus affected the age to be younger, but this question requires further investigations.

Hypertension would be the result of the older age. However, because the sample size is not very large, and the significance was marginal, we suggest larger studies to reveal the significant relationships.

The strengths of our study are threefold. First, we conducted intereye comparison of risk factors where individual variability of systemic and demographic factors can be strictly controlled. Second, the MD difference cutoff was very large compared to previous studies. This marked difference would have led to clearer results. Third, we recruited symmetric NTG patients as a control group and the absence of significant differences in parameters strengthened our conclusions.

This study carries several limitations. First, it was a retrospective review, and was not designed to evaluate cause-and-effect relationship. But with this large difference of MD (6dB, an interval unit to classify the VF defect as mild, moderate, and severe) it is not likely that the two eyes have had comparable velocity of deterioration. In other words, the worse eye would likely have had a faster glaucoma progression. In addition, the cohort size is not small, and our speculation would benefit from the design of intereye comparison because effects of confounding factors can be eliminated enabling evaluation of pure influences of ocular parameters only.

Second, the degree of myopia was evaluated only through refractive error, not axial length. But within the same patient, the keratometric values, chamber depth, and status of lens would be correlated and therefore, refraction could reliably represent the relative status of axial myopia. Also, we did not investigate the microstructures of PPA. We believe those characteristics should be explored further.

Third, the MD slopes were not analyzed. We did not analyze the rate of progression as change of MD per year, which is an indispensable parameter when discussing the velocity of glaucoma deterioration. Nevertheless, eliminating inter-individual variability can greatly facilitate precise and clear comparison. Our study also benefits from minimal inter- and intra-individual variabilities on VF tests. Besides, MD slope itself changes with natural course of disease. Therefore, although only assumptions can be drawn from this cross-sectional study, we considered that it is not flawed to confer the relative susceptibility for glaucoma deterioration with our methods.

It is also possible that earlier development of glaucoma in one eye have made the more severe VF result. Nevertheless, 6 dB of MD difference cannot be rapidly achieved in a short period of time. We excluded every detectable case with considerable time gap between the onset of glaucoma between the two eyes. Nevertheless, we were not able to trace all the cases for the beginning of glaucomatous of RNFL damage. It is difficult to separate the issue of glaucoma onset and progression because the parameters could have fastened the onset of glaucoma, and at the same time accelerated the progression of it. Only longitudinal studies would provide helpful information on this issue. Effect of treatment was eliminated by restricting patients with identical treatments in both eyes.

Finally, the results are confined to NTG patients of Korean population. It remains an open question whether our results could be applied to POAG patients, and other ethnicities. It has been proposed that NTG and POAG be considered as a single disease spectrum,[78] but cautions are required.

In conclusion, we could observe through intereye comparisons that the more myopic eye in myopic NTG, and the more pressured eye in nonmyopic NTG demonstrates more severe VF. PPA was also a significant factor, but clinical interpretation remains speculative. Myopic and nonmyopic patients may show different patterns of pathologic processes. The evaluation of bilateral NTG patients on the susceptibility of deterioration should be approached discriminately upon different subgroups. Further investigations on microstructure of the ONH are warranted.

## Supporting information

S1 Fig Distribution of age mean deviation in the study population. Graphs are provided for asymmetric group and symmetric group, respectively.
(XLSX)

S2 Fig Distribution of mean deviation in the study population. Graphs are provided for worse eye and better eye, and for asymmetric group and symmetric group, respectively.
(XLSX)
